# Peptide-Based Vaccination Therapy for Rheumatic Diseases

**DOI:** 10.1155/2020/8060375

**Published:** 2020-03-18

**Authors:** Bin Wang, Shiju Chen, Qing Zheng, Yuan Liu, Guixiu Shi

**Affiliations:** Department of Rheumatology and Clinical Immunology, The First Affiliated Hospital of Xiamen University, Xiamen 361003, China

## Abstract

Rheumatic diseases are extremely heterogeneous diseases with substantial risks of morbidity and mortality, and there is a pressing need in developing more safe and cost-effective treatment strategies. Peptide-based vaccination is a highly desirable strategy in treating noninfection diseases, such as cancer and autoimmune diseases, and has gained increasing attentions. This review is aimed at providing a brief overview of the recent advances in peptide-based vaccination therapy for rheumatic diseases. Tremendous efforts have been made to develop effective peptide-based vaccinations against rheumatoid arthritis (RA) and systemic lupus erythematosus (SLE), while studies in other rheumatic diseases are still limited. Peptide-based active vaccination against pathogenic cytokines such as TNF-*α* and interferon-*α* (IFN-*α*) is shown to be promising in treating RA or SLE. Moreover, peptide-based tolerogenic vaccinations also have encouraging results in treating RA or SLE. However, most studies available now have been mainly based on animal models, while evidence from clinical studies is still lacking. The translation of these advances from experimental studies into clinical therapy remains impeded by some obstacles such as species difference in immunity, disease heterogeneity, and lack of safe delivery carriers or adjuvants. Nevertheless, advances in high-throughput technology, bioinformatics, and nanotechnology may help overcome these impediments and facilitate the successful development of peptide-based vaccination therapy for rheumatic diseases.

## 1. Introduction

Rheumatic diseases consist of more than 100 heterogeneous autoimmune disorders and can result in substantial morbidity and mortality [1]. The pathogenic mechanisms of most rheumatic diseases have not been clearly defined. Apart from the physical impairment, rheumatic diseases also have caused a heavy socioeconomic burden [2, 3]. The treatment of rheumatic diseases such as rheumatoid arthritis (RA), systemic lupus erythematosus (SLE), and Sjögren's syndrome (SjS) varies across patients with different clinical characteristics [4–6]. Current therapies for rheumatic diseases mainly include conventional synthetic disease-modifying antirheumatic drugs (DMARDs) and newly developed biologic therapies [4, 7]. The introduction of biologic therapies has revolutionized the treatment of many rheumatic diseases such as RA and ankylosing spondylitis (AS) in the past decade. However, a large part of patients with rheumatic diseases are still not well treated, which is possibly attributed to poor response to therapeutic agents, delayed diagnosis, or poor medication adherence [4, 7, 8]. Additionally, biologic therapies such as tumor necrosis factor-*α* (TNF-*α*) antagonists (monoclonal antibodies or soluble receptors) can increase the risk of opportunistic infections such as tuberculosis [9]. Moreover, the clinical application of biological agents is still limited for their high costs especially in developing countries [10]. Therefore, there is a pressing need in the development of both more safe and cost-effective treatment strategies for rheumatic diseases.

As the greatest success in public health, the major goal of vaccination is to prevent infections such as influenza, tuberculosis, hepatitis, and malaria [11, 12]. Nevertheless, the roles of vaccinations in the treatment of noninfection diseases such as cancer and allergic diseases have gained increasing attentions in recent years [13–17]. Among those distinct approaches of therapeutic vaccines, peptide-based vaccination is a highly desirable strategy and has gained increasing attentions [18, 19]. Peptide-based vaccines are aimed at precisely inducing immune response against antigens by key epitope peptides but not the entire antigen and thus have several advantages over traditional vaccines such as higher specificity, higher safety, lower costs, and less adverse events [19–21]. Studies in recent years have suggested that peptide-based vaccinations are promising in treating diseases such as cancer and allergic diseases, and some have shown impressive clinical benefits [22–24]. Besides, peptide-based vaccination has also been proposed as a promising immunotherapy for autoimmune diseases such as type 1 diabetes mellitus (T1DM) and multiple sclerosis (MS) [25–27]. Some studies also have reported encouraging findings on peptide-based immunotherapeutic vaccinations in rheumatic diseases [28–32]. This review is aimed at providing a brief overview of recent advances in peptide-based vaccinations in rheumatic diseases such as RA, SLE, and SjS.

## 2. Types of Peptide-Based Vaccination Therapy

The pathologic hallmark of rheumatic diseases is the breakdown of immune homeostasis and the loss of immune tolerance to self-antigens, which can trigger the formation of autoreactive T cells and B cells recognizing epitopes on autoantigens [33]. Both autoreactive immune cells and their secreted cytokines can result in harmful autoreactive immune attacks towards host cells and tissues, and thus, they are the two main targets for immunotherapy in rheumatic diseases. Distinct forms of peptide-based vaccinations have been studied for therapeutic or preventive strategies for diseases such as cancer, rheumatic diseases, and allergic disorders [34–38]. According to the therapeutic targets, peptide-based vaccinations used for rheumatic diseases can be classified into two main subtypes including peptide-based active vaccination against pathogenic cytokines and peptide-based tolerogenic vaccination. The former mainly targets pathogenic cytokines, while the latter mainly targets autoimmune attacks against host cells or tissues and is aimed at inducing immune tolerance by inhibiting autoreactive lymphocytes (Table 1).

### 2.1. Peptide-Based Active Vaccination against Pathogenic Cytokines

Pathogenic cytokines such as TNF-*α*, interferon-*α* (IFN-*α*), and interleukin-6 (IL-6) are critical mediators of autoimmune damages to host cells and tissues and have long been regarded as therapeutic targets for rheumatic diseases [39]. Passive immunization aimed at neutralizing the pathogenic cytokines such as TNF-*α* and IL-6 with monoclonal antibodies (mAbs) or soluble receptors has been proven to be an effective therapy strategy of rheumatic diseases such as RA and AS [5]. However, passive immunization with mAbs targeting cytokines has several drawbacks such as risk of infections, antidrug antibodies, high cost, and low treatment response, which suggests the need of an alternative therapeutic approach to target those cytokines [40, 41]. Therapeutic active vaccination against pathogenic cytokines has been proposed to be a promising treatment strategy in treating rheumatic diseases and has gained increasing concerns in recent years [42–44]. Compared with passive immunization therapy, therapeutic active vaccination has several possible advantages such as lower costs, lower risk of infections, and less frequent administrations.

Active vaccination with the entire molecule or key peptides derived from targeted cytokines can elicit the activation of B cells and trigger the production of neutralizing antibodies against pathogenic cytokines, thus inhibiting the pathogenic effects of those cytokines [45]. Distinct forms of engineered immunogens have been used such as entire inactive molecule, key epitope peptides, modified peptides, or engineered DNA vaccine encoding pathogenic molecules. Moreover, vaccines containing multiepitope peptides may also be considered, which may restore wider immune tolerance and achieve more benefits than a single peptide-based vaccination. Active immunization with entire pathogenic molecules or key peptides can both induce neutralizing antibodies against those pathogenic molecules, but the former have a higher risk of inducing nonneutralizing antibodies or cross-reactive antibodies against other host self-antigens. Therefore, peptide-based vaccinations are more promising to be used in clinical practice since they can induce peptide-specific antibodies and decrease the risk of cross-reactivity.

A widely studied therapeutic active vaccination is the active immunization against TNF-*α*, which has been proposed as a promising alternative strategy for TNF-*α*-targeting therapy. Previous studies have reported a successful vaccination therapy using a compound named kinoid of human TNF-*α* (TNF-K) in the treatment of RA [46, 47]. TNF-K contains the entire inactivated human TNF-*α* and keyhole limpet hemocyanin (KLH) as a carrier protein. Though TNF-K is not a peptide-based vaccine, it has been proven to be a successful active vaccination against the pathogenic TNF-*α* in RA in both preclinical studies and clinical trials and has thus provided some indications for future studies exploring the feasibility of peptide-based anti-TNF-*α* active vaccination in treating rheumatic diseases [48, 49]. Moreover, numerous studies have explored distinct forms of peptide-based active vaccinations against TNF-*α* and also have provided encouraging findings in experimental studies using animal disease models.

Anticytokine active vaccination needs to overcome the natural tolerance of the immune system to self-proteins and thus induce high titers of effective neutralizing antibodies. However, a major shortcoming for peptide-based vaccines is the low immunization response caused by minimal antigenic epitopes, which is a major limitation during the development of an effective anticytokine active vaccination for rheumatic diseases. To ensure the immunization response or the efficacy of peptide-based vaccines, adjuvant or other molecules with adjuvant potency is especially necessary. Most previous studies using animal models used traditional adjuvants, while other studies used some carrier molecules to increase the immunogenicity of peptides such as virus-like particles (VLPs) [50–52]. VLPs can induce potent B cell responses effectively even in the absence of adjuvants and thus can be used in the molecular assembly system to induce strong B cell responses against most antigens [50]. Currently, there is a lack of both effective and safe adjuvants to ensure the use of peptide-based vaccinations in clinical trials, which is also a major obstacle in limiting the clinical use of peptide-based anticytokine active vaccination in treating rheumatic diseases. Considering the autoimmune reaction risk caused by some adjuvants [53], adequate adjuvants or carrier molecules with both high capability of inducing immune response and high safety are urgently needed for the clinical use of peptide-based vaccines. Advances in vaccine design technology such as the promising nanoparticle-carried vaccines may help overcome this limit.

### 2.2. Peptide-Based Tolerogenic Vaccinations

Rheumatic diseases are characterized by the breakdown of immune homeostasis and loss of immune tolerance to self-antigens, which further triggers the formation of autoreactive lymphocytes and autoimmune attacks to host tissues [54, 55]. Therefore, rebalancing immune homeostasis by inducing immune tolerance is a critical strategy in treating rheumatic diseases [33, 56]. Compared with conventional immune suppression therapy and biologic agents, immune tolerance induction therapy has the potential to inhibit autoimmune attacks while at the same time maintaining the ability to cope with danger signals, leading to a safe and efficacious therapy for rheumatic diseases [56]. Several strategies of inducing immune tolerance have been proposed as candidate treatments for rheumatic diseases such as stem cell therapy, tolerogenic dendritic cells (DCs) therapy, expansion of T regulatory cells (Tregs) by low-dose IL-2, and tolerogenic vaccination therapy [57–59]. Among them, treating rheumatic diseases through peptide-based tolerogenic vaccination is of great interest and has gained increasing concerns in recent years [28, 60, 61].

It has been well defined that vaccination with an entire antigen or key tolerogenic peptides in the absence of adjuvant or costimulation signals has the potential to induce antigen-specific immune tolerance, which is a potentially effective approach in treating autoimmune diseases [62–64]. Therefore, modulation of the pathogenic immune response through antigen-specific tolerogenic vaccination has the potential to restore immune tolerance and ameliorate autoimmune attacks in rheumatic diseases [62, 65]. Most of those studies were based on animal models of autoimmune diseases, while relevant clinical studies are still limited. Several clinical trials had evaluated the safety and feasibility of peptide-based tolerogenic vaccination in patients with autoimmune diseases such as T1DM, RA, and MS, and some of them showed encouraging findings [66, 67]. Unlike the vaccines against infections which contain non-self-antigens and are aimed at inducing active immunization, tolerogenic vaccines contain self-antigens or key peptides and are aimed at inducing antigen-specific immune tolerance [28, 56]. Moreover, contrary to the capability of peptide-based anticytokine active vaccination in eliciting a strong immune response and inducing the activation of autoimmune B cells, peptide-based tolerogenic vaccinations are aimed at reestablishing immune tolerance to eliminate attacks.

The selection of epitope peptides for tolerogenic vaccinations is a critical essential step. Some antigen epitopes may mainly exert roles in the development of rheumatic diseases as immunogens to induce autoimmune response, while others may mainly act as tolerogens to induce immune tolerance [68–71]. Some antigens may have the capability of inducing either an immune response or immunologic tolerance under different exposure conditions and concomitant stimulators. Therefore, epitope peptides with the potential to induce immune tolerance under different exposure conditions are ideal targets for tolerogenic vaccinations. However, the ideal candidate epitope peptides for most rheumatic diseases are still largely elusive. Advances in vaccinomics and immunoinformatics may promote the identification of T and B cell epitopes by integrating useful information from multiple databases of different disciplines [72–75]. Moreover, both native and posttranslational modified epitopes have the possibility of exerting critical roles during the development of autoimmunity, both of which have the potential to be candidate therapeutic targets for tolerogenic vaccinations. Apart from epitope peptides from self-antigens, analog peptides of epitopes produced mainly by amino acid substitutions also have the potential to be candidate tolerogenic peptides [76]. There are also some tolerogenic peptides with therapeutic potential for rheumatic diseases, though they are not the peptides of certain antigens involved in the pathogenesis of diseases. hCDR1 is a tolerogenic peptide designed by the sequence of the heavy chain complementarity-determining region 1 (CDR1) of monoclonal anti-DNA antibodies and has been proven to be able to treat SLE by peptide-specific induction of Tregs [77–79].

Though the molecular mechanisms underlying the effects of peptide-based tolerogenic vaccinations in treating autoimmune diseases is still not clearly defined, their roles in mediating the anergy of autoactive T cell and promoting the expansion of Tregs have been considered to be major contributors [80, 81]. Tolerogenic peptides can be taken up by antigen-presenting cells (APCs) such as DCs, which further induce immune tolerance by inhibiting autoactive T cell or inducing Tregs. Recent studies reveal that central tolerance mediated by negative selection can prune but not completely eliminate autoreactive T cells, which leads to the incomplete negative selection and the existence of autoreactive T cells in the circulating system among healthy individuals [82–85]. The findings above further suggest the importance of peripheral tolerance in fighting against autoimmunity such as Treg-mediated suppression and the necessity of reestablishing immune tolerance by tolerogenic vaccinations in treating autoimmune diseases.

DCs are key immune cells which not only present antigens to adaptive immune cells such as T cells but also have a critical role in regulating immune tolerance [86]. Inadequate activation of DCs can cause autoimmunity by inducing the activation and differentiation of autoreactive T cells or B cells. However, the induction of tolerogenic DCs (tolDCs) with tolerogenic features and the ability of ameliorating autoimmunity have emerged as a promising therapy for autoimmune diseases [87]. tolDCs can produce anti-inflammatory cytokines and deviate T cells to regulatory or immunosuppressive phenotypes, thus inhibiting autoreactive T cells [88]. Currently, an alternative approach to induce antigen-specific immune tolerance is the induction of tolDCs towards self-antigens [33, 89]. tolDCs may present antigens to T cells but not give strong costimulatory signals owing to the low expression levels of costimulators, which can lead to the deletion or anergy of autoreactive T cells and induce Tregs. Antigen-boosted tolDCs have been proposed as a promising approach in treating autoimmune diseases [90]. Some clinical studies have been done to evaluate the safety and efficacy of autologous tolDCs loaded with autoantigens or key peptides in treating rheumatic diseases. Nevertheless, the adequate selection of epitope peptides from autoantigens for the induction of tolDCs is also critical for the efficacy of this intervention strategy [91].

With the rapid advances in nanotechnology, nanoparticle-carried vaccines have emerged as novel approaches to vaccine design, and their use in peptide-based tolerogenic vaccinations has gained increasing concerns in recent years [19, 92]. Nanoparticles coated with tolerogenic antigen peptides is a novel and promising strategy for inducing antigen-specific immune tolerance, which may promote the application of peptide-based vaccination in rheumatic diseases. Those nanoparticles can be taken up by APCs such as DCs, which further induce immune tolerance by mediating the anergy of autoactive T cell and promoting the expansion of Tregs. Several recent studies have revealed that vaccinations with nanoparticles carrying peptides can induce antigen-specific immune tolerance and represent a potential approach for the treatment of autoimmune diseases [93–95]. For instance, a recent study by Clemente-Casares et al. reported that a systemic therapy with nanoparticles coated with disease-specific peptides could trigger immunosuppressive immune cells, such as antigen-specific regulatory T cell type 1- (TR1-) like cells and regulatory B cells, and suppress autoantigen-loaded APCs in mouse models of T1DM, MS, and RA, which was a potential treatment for autoimmune diseases [94].

Apart from peptides from self-antigens, those from T-cell receptor (TCR) also have been explored as therapeutic vaccines to treat autoimmune diseases including rheumatic diseases, which may be mediated by their roles in modulating autoreactive T cells or activating Tregs [96–99]. Some studies have provided encouraging findings regarding the safety and the efficacy of TCR peptide-based therapeutic vaccines in patients with rheumatic diseases [96]. However, the molecular mechanisms underlying the therapeutic roles of TCR peptide-based vaccines are still not clearly defined and further studies are needed on this aspect. The advances in the technologies to assess TCR repertoire have provided much help in precisely identifying dominant TCR repertoire involving the development of rheumatic diseases, which may further facilitate the development of TCR peptide-based therapeutic vaccines for those diseases [100, 101].

## 3. Peptide-Based Vaccinations for Rheumatic Diseases

Numerous studies have been carried out to evaluate the feasibility of peptide-based vaccinations in treating rheumatic diseases, but most of them are related to RA and SLE and several studies focus on SjS. Therefore, the advances of peptide-based vaccinations in RA, SLE, and SjS are reviewed in detail in the following part, and the other rheumatic diseases are not referred owing to the lack of relevant studies.

### 3.1. RA

RA is a common rheumatic disease affecting joints which is characterized by inflammatory synovitis, progressive bone erosion, and joint destruction [102]. Improved understanding of RA pathogenesis has led to the development of several effective targeted biological treatments. Although conventional and biological antirheumatic drugs can substantially reduce disease activity and inflammation, many RA patients are still inadequately managed and suffer from unfavorable treatment outcomes [7, 102]. Therefore, to further improve the treatment outcomes of RA patients, more new therapeutic strategies are urgently needed. Peptide-based vaccination has been suggested to be a promising treatment strategy for RA.

Several key pathogenic molecules involved in the pathogenesis of RA have been identified, and targeting those molecules with passive immunotherapy have been proven to be effective in RA, such as TNF-*α* and IL-6. As a proinflammatory cytokine, TNF-*α* has an essential role in the pathogenic process of RA and is a well-validated target. Studies on therapeutic active vaccinations against pathogenic cytokines also have mainly aimed at targeting TNF-*α* [103–105]. Some experimental studies have explored whether active immunization with peptide-based vaccines against TNF-*α* could ameliorate autoimmune arthritis in animal models of RA. Capini et al. reported that active immunization with TNF-*α* peptides could generate endogenous autoantibodies against TNF-*α* [106]. Chackerian et al. revealed that vaccination of mice with conjugated particles containing VLPs and TNF-*α* peptides could generate autoantibodies against TNF-*α* and inhibit the development of collagen-induced arthritis (CIA) [107]. Another study by Spohn et al. found that VLP-based TNF-*α* peptide vaccine could trigger specific antibodies and ameliorate arthritis signs without inducing reactivation of latent tuberculosis [108]. Zhang et al. designed a TNF-*α* epitope-scaffold immunogen using the transmembrane domain of diphtheria toxin, which could induce sustained neutralizing antibodies against TNF-*α* and alleviate CIA in mice [109]. Another study reported that a dual-targeting vaccine using two segments of the TNF-like domain of activator of the NF-kB ligand (RANKL) linked to the peptide EWEFVNTPPLV could induce neutralizing antibodies against TNF-*α* and RANKL and thus could ameliorate both bone destruction and inflammation severity by simultaneously inhibiting TNF-*α* and RANKL [110].

Interleukin-1*β* (IL-1*β*), IL-6, vascular endothelial growth factor (VEGF), and IL-23 are crucial cytokines involved in the pathogenesis of RA. Bertin-Maghit et al. reported that synthetic IL-1*β* peptides could lead to autoantibodies against IL-1*β*, thus inhibiting the inflammation and articular destruction in CIA mice [111]. Moreover, vaccination with IL-6 analogs could induce autoantibodies to IL-6 and protect against CIA [112]. Semerano et al. found that a peptide derived from VEGF linked to the KLH carrier protein could ameliorate inflammation and joint destruction in experimental arthritis by inducing neutralizing anti-VEGF Abs [113]. Ratsimandresy et al. found that a murine IL-23p19 peptide predicted by bioinformatics could trigger anti-IL-23 antibodies and induce protection against joint destruction and inflammation in CIA mice [114].

Apart from peptide-based vaccination against pathogenic cytokines, peptide-based tolerogenic vaccinations have been proven to be successful in the prevention and treatment of arthritis in animal models [29, 115]. Type II collagen (CII) is a well-defined autoantigen for RA and has been widely used to induce animal models of RA [116]. A study by Myers et al. revealed that an epitope peptide from CII cyanogen bromide 11 (CB11) fragment p122-147 could suppress autoimmune arthritis by inducing immune tolerance in a mouse model of CIA [117]. Another study by Ku et al. reported that vaccination with an immunodominant epitope peptide from CII CB11 p58-73 could prevent experimental arthritis in either neonatal or adult rats [118]. Several other studies further reported that administration of CII immunodominant peptides such as p184-198, p181-209, and p245-270 could suppress autoimmune response and ameliorate arthritis in CIA animal models by their tolerogenic effects [119–123]. Apart from the original CII peptides, various analog peptides of CII immunodominant peptides have also been shown to suppress autoimmune arthritis by inhibiting autoimmune T cell responses and inducing immune tolerance [124–129]. Some studies also had explored the use of vaccine delivery systems in the CII peptide-based therapeutic vaccinations for RA. Zimmerman et al. found that a Ligand Epitope Antigen Presentation System (LEAPS) therapeutic vaccine containing a human CII peptide could modulate autoimmune response and reduce disease progression in the CIA mice [130]. Mikecz et al. reported that the proteoglycan (PG) immunodominant peptide PG70 attached to either DerG (DerG-PG70) or J immune cell-binding peptide (J-PG70) through LEAPS could suppress arthritis through reducing pathogenic T cell responses and promoting immunosuppressive T cells in two mouse models of RA [30].

Heat-shock proteins (HSPs) are a possible source of autoantigens from stressed cells or inflamed tissues in autoimmune diseases, and several peptides from HSPs such as HSP60, HSP65, or HSP70 have been proven to ameliorate autoimmunity in animal models of RA [131–135]. Prakken et al. reported that vaccination with HSP60 peptide containing a T cell epitope could suppress avridine-induced arthritis in rats [134]. Studies by Zonneveld-Huijssoon et al. revealed that microbial HSP60 peptide vaccine could prevent experimental arthritis by enhancing Tregs [135, 136]. A HSP70 epitope peptide B29 was found to be able to induce the protective Tregs and suppress arthritis in mice [133, 137, 138], while autologous tolDCs loaded with HSP70 B29 peptide may be a candidate therapy for RA [70]. Studies by Moudgil et al. found that pretreatment with peptides comprising mycobacterial heat-shock protein 65 (BHSP65) carboxy-terminal determinants but not the amino-terminal determinants could suppress the development of arthritis in Lewis rats [139–141]. In RA patients, a peptide derived from a heat-shock protein of bacteria (dnaJP1) administered orally significantly increased the percentage of T cells producing IL-4 and IL-10 and reduced TNF-*α* [131]. Several other studies also have found that some peptides derived from HSPs could inhibit autoimmune arthritis [142–144].

Antibodies against citrullinated proteins such as filaggrin, vimentin, and collagen type II have crucial roles in the pathogenesis of RA [145, 146]. Prophylactic administration of a citrullinated filaggrin peptide could reduce disease severity and incidence of arthritis in a CIA animal model [145]. Another study by Gertel et al. reported that vaccination with multiepitope peptides derived from citrullinated autoantigens could induce immune tolerance and attenuate arthritis manifestations by promoting Treg cells and inhibiting Th17 cells in an animal model of RA [147]. A further study by Gertel et al. found that a multiepitope peptide derived from citrullinated autoantigens could modulate both the expressions of key cytokines and the frequencies of T cells in peripheral blood mononuclear cells (PBMCs) from RA patients [148].

A promising immunotherapy aimed at restoring self-tolerance is the induction of antigen-specific tolerance by tolerogenic immune cells loaded with autoantigens or tolerogenic nanoparticles loaded with pathogenic peptides [149]. A phase 1 trial by Bell et al. revealed that intraarticular injection of autologous tolDCs loaded with autoantigens from autologous synovial fluid could be a safe and feasible therapy for RA patients [150]. Another phase 1 trial by Benham et al. revealed that intradermal injection of autologous modified DCs exposed to citrullinated peptides could increase the ratio of regulatory to effector T cells and reduce inflammatory cytokines in HLA risk genotype-positive RA patients [151].

Apart from peptides from autoantigens, TCR peptides also have been proposed as promising therapeutic vaccines for RA. Some studies using animal models of RA found that vaccination with TCR V beta chain peptides could prevent CIA by inhibiting pathogenic T cells [152]. Some clinical studies also have provided encouraging findings regarding the safety and the efficacy of TCR peptide-based therapeutic vaccines in RA patients [96, 153]. A placebo-controlled trial reported by Moreland et al. found that vaccination with a combination of Vbeta3, Vbeta14, and Vbeta17 TCR peptides was well tolerated and was effective in RA patients [153].

Previous studies on peptide-based vaccinations for RA have reported encouraging findings. However, most studies available now have been mainly based on animal models, while evidence from clinical studies is still limited. More studies are urgently needed to facilitate the development of an effective and safe peptide-based vaccination for RA patients. In addition, though immune tolerance induction with peptide-based vaccinations have been proven to be effective in treating RA, the underlying molecular mechanisms are still largely elusive and need to be elaborated in further studies.

### 3.2. SLE

SLE is a devastating and heterogeneous rheumatic disease affecting multiple organs such as the skin, hematopoietic system, and kidney [154]. Apart from those conventional drugs such as immunosuppressants and corticosteroids, the advances in targeted biological agents have substantially improved the prognosis of SLE patients [155]. However, adequate control of disease activity or achieving remission is still challenging for a large part of SLE patients, and those patients are at high risk of premature mortality [4]. Therefore, more innovative treatment strategies need to be developed to improve the prognosis of SLE patients. Some studies have explored peptide-based therapeutic vaccinations as potential therapies for SLE, some of which have uncovered promising outcomes.

Some studies have explored the feasibility of active vaccination against pathogenic cytokines such as IFN-*α* in the treatment of SLE. IFN-*α* has long proven to be a major pathogenic cytokine in the pathogenesis of SLE [156]. Anti-IFN-*α* drugs such as anifrolumab and sifalimumab have been shown to substantially reduce disease activity in patients with moderate-to-severe SLE [157–159]. Mathian et al. found that active immunization of human IFN-*α* transgenic mice with a human IFN-*α* kinoid (IFN-K) could induce polyclonal neutralizing antibodies against IFN-*α*, suggesting that IFN-K vaccination may be a promising therapy for SLE [160]. IFN-K vaccine could effectively ameliorate lupus manifestations by inducing neutralizing antibodies in both mouse lupus model and SLE patients [160–163]. Clinical trials showed that IFN-K was well tolerated and significantly reduced disease activity in SLE patients [163, 164]. Vaccination therapy by targeting pathogenic cytokines such as IL-17 has also been studied as potential treatments for SLE [161, 165, 166]. B cell-targeted therapy has been regarded a promising therapeutic approach for SLE, and anti-CD20 monoclonal antibodies such as rituximab have been proven to be effective in SLE patients. Active immunization with a CD20 mimotope peptide could induce B cell depletion and increase survival in a mouse SLE model, which offered an alternative approach for B cell depletion therapy [31].

Apart from peptide-based vaccination against pathogenic cytokines, peptide-based tolerogenic vaccinations have also been studied as a candidate treatment for SLE. Many peptide autoepitopes have been proven to be involved in the pathogenesis of SLE [167–169]. Some histone peptides such as histone H4 autoepitope peptide 16-39 (H4_16-39_) and autoepitope peptide 71-94 (H4_71-94_) could induce an inflammatory response, whereas others such as H2A_34-48_ could lead to an immunosuppressive response [167, 168]. Therefore, different peptides can lead to distinct immune response during the development of SLE. A study using human PBMC cultures found that a mixture of histone autoepitope peptides could block pathogenic autoimmune response and restore immune homeostasis in lupus [170]. Treatment with H4_16-39_ could delay the onset of severe lupus nephritis possibly by the tolerogenic effect on autoimmune Th cells and autoimmune B cells in a mouse model of lupus [167]. Other studies found that treatment with H4_71-94_ could suppress pathogenic lupus T cells by inducing regulatory T cells [171, 172]. Several other studies also had shown that peptides derived from histone proteins could suppress murine lupus by inducing immune tolerance [173–176]. Additionally, some peptides derived from other self-antigens have also been explored as candidate therapeutic vaccines for SLE. For instance, a phosphorylated spliceosomal epitope, the P140 peptide, could repress B cell differentiation and ameliorate lupus [169, 177, 178]. Subsequent clinical studies further showed that the P140 peptide could improve the clinical and immune status of SLE patients [179–181].

Peptides from other sources such as anti-DNA mAbs have also been explored as candidate therapeutic vaccines for SLE [182, 183]. Singh et al. found that a peptide from the variable regions of heavy chains of anti-DNA mAbs could delay the onset of autoimmunity in a lupus mouse model by inducing immune tolerance [182]. Waisman et al. reported that peptides from CDRs of pathogenic anti-DNA mAbs could prevent autoantibody production and downregulate autoreactive T cell responses, representing a potential treatment for SLE [184, 185]. Several other studies further revealed that a tolerogenic peptide derived from the CDR1 of a human anti-DNA autoantibody (hCDR1) could ameliorate lupus by inducing Tregs and suppressing the activation of autoreactive cells in lupus animal models [186, 187]. Several possible mechanisms have also been proposed to explain the therapeutic role of hCDR1 in SLE, such as TGF-*β*-mediated suppression of autoreactive T cells and downregulation of transcription factors responsible for negative regulation of T cell activation [188, 189]. Based on the encouraging findings from experimental studies, several clinical studies were done to assess the efficacy and safety of hCDR1 (Edratide) in SLE patients, which revealed favorable outcomes in SLE patients receiving hCDR1 treatment [190–192]. Another artificial peptide pConsensus (pCons) based on the immune determinants of anti-DNA IgG sequences has also been shown to be effective in delaying disease onset in the lupus mouse model by inducing immune tolerance and promoting Treg activity [193–197].

Though many studies had explored the possible roles of peptide-based vaccinations in treating SLE, most of them were experimental studies using animal models and few were clinical studies. The efficacy of safety of peptide-based vaccinations in SLE patients need to be explored by future clinical trials. In addition, though some pathogenic autoantibodies have been well characterized for SLE, the useful peptides for vaccination therapy of SLE are still not well defined. Future studies exploring candidate peptides for the effective vaccination therapy in SLE are recommended.

### 3.3. SjS

SjS is a complex and heterogeneous rheumatic disease characterized by exocrinopathy, severe fatigue, and various systemic manifestations [6]. The treatment options currently available for SjS patients are still limited especially for those with extraglandular diseases, and more studies are needed to expand the treatment options [198]. Some efforts have been made to explore the feasibility of peptide-based vaccinations for SjS in the past decade, and some have provided encouraging findings.

HSP60 and muscarinic acetylcholine 3 receptor (M3R) are important autoantigens involved in the pathogenesis of SjS [199–202]. A study by Delaleu et al. reported that vaccination with a HSP60-derived peptide (aa 437-460) could significantly reduce SjS-related histopathologic features and retain normal exocrine function in nonobese diabetic (NOD) mice [203]. Yang et al. found that a M3R peptide (aa 208-227) immunization could reduce cytokines, such as IL-17 and IFN-*γ*, and inhibit lymphocytic infiltration in mice [204]. An in vitro experiment by Sthoeger et al. revealed that the tolerogenic peptide hCDR1 could significantly reduce the expressions of IL-1*β* and TNF-*α* but increase the expressions of TGF-*β* and FOXP3 in the PBMCs of SjS patients, suggesting hCDR1 as a potential candidate treatment for SjS [205]. Another study by Li et al. found that the P140 peptide generated from a spliceosomal protein could rescue MRL/lpr mice from immune infiltration and autophagy defects in the salivary glands, suggesting a candidate therapy for SjS [206].

Currently, there is no study investigating the role of peptide-based vaccination against pathogenic cytokines in the treatment of SjS. Several pathogenic cytokines have been identified in the development of SjS such as IFN-*α* and IL-17, and further studies are recommended to evaluate the feasibility of peptide-based vaccination against these pathogenic cytokines in treating SjS [207–209]. Moreover, studies on peptide-based tolerogenic vaccinations in the treatment of SjS are also limited, and more studies are recommend to explore them.

## 4. Conclusions and Perspectives

Current therapies for most rheumatic diseases are mainly aimed at ameliorating symptoms and control disease progression, and there is still a pressing need in developing more safe and cost-effective treatment strategies. Peptide-based vaccination therapy is a highly desirable and curative strategy in treating rheumatic diseases and has the potential to revolutionize the therapy of rheumatic diseases. Though tremendous efforts from previous studies have been made to develop effective peptide-based vaccinations against rheumatic diseases such as RA and SLE, most studies have been done using animal models while evidence from clinical studies is still limited. Additionally, the roles of peptide-based vaccinations in other rheumatic diseases such as AS are still largely elusive and thus need to be determined by more studies in the future.

Despite encouraging findings from studies using animal models, only a few clinical trials have been done to assess their clinical benefits, and some of them have failed to replicate the promising findings from experiment studies using animal disease models. A major obstacle is the differences between animals and humans in both immune response and immune tolerance, and findings from animal models are frequently not applicable to humans. A precise identification of those pathogenic antigens and key epitopes which exert roles in both animal models and humans may help to facilitate the studies of peptide-based vaccines. Additionally, disease heterogeneity is a well-defined characteristic of rheumatic diseases, and the immunodominant pathogenic epitopes are different across patients with distinct disease stages or clinical characteristics, which can limit the therapeutic efficacy of vaccinations targeting a small part of epitopes. Neoepitopes originating from epitope spreading or modified epitopes can further increase disease heterogeneity [65]. Therefore, personalized peptide vaccinations may be a more adequate approach for developing effective vaccination therapy against rheumatic diseases, in which peptides for targeted vaccinations are specifically selected for each individual patient. Finally, an unignored obstacle is the lack of both effective and safe adjuvants for the use of peptide-based vaccinations in clinical trials. Advances in vaccine design technology such as the promising nanoparticle-carried vaccines may help to overcome this limit [94, 210, 211].

The precise identification of immunodominant epitopes or neoepitopes from pathogenic cytokines or autoantigens is critically important for the successful development of peptide-based vaccinations in treating rheumatic diseases. Recent advances in high-throughput technology, vaccinomics, and bioinformatics have helped us in identifying key immunodominant epitopes from pathogenic cytokines and essential epitope peptides from autoantigens as promising targets for the peptide-based vaccinations [65, 212]. The proper implementation of computational prediction tools of bioinformatics may facilitate the development of more innovative and effective peptide-based vaccines for rheumatic disease and also may promote the translation from preclinical studies to clinical trials. Besides, the pathogeneses of most rheumatic diseases are still not clearly defined, and dominant self-antigens involved in disease development have not yet been identified. Further studies are still urgently needed to expand our understanding of the pathogeneses of rheumatic diseases, which may uncover new therapeutic targets or dominant antigens and pave new avenues for peptide-based vaccinations for the treatment of those diseases [213].

A careful screening of epitope peptides is a critical prerequisite for the efficacy and safety of peptide-based vaccination therapy in autoimmune diseases including rheumatic diseases [214, 215]. During the development of rheumatic diseases, epitope specificity exists in the pathogenic roles of antigens or cytokines, and different epitope peptides thus can exert obviously distinct roles in modulating immune response [216, 217]. Some epitope peptides can precipitate but not inhibit disease progression. For most rheumatic diseases, an antigen epitope able to induce immunologic tolerance is still largely elusive and it is a major challenge in developing peptide-based tolerogenic vaccinations for rheumatic diseases.

With the complex autoimmune networks, diverse autoantigens, and distinct autoreactive T cells usually exist in patients with rheumatic diseases such as SLE, SjS, and RA. Multiple autoantigens contribute to the pathogeneses of these rheumatic diseases and targeting those autoantigens separately may only have limited therapeutic potential. Therefore, treatment with a complex of epitope peptides from multiple autoantigens may increase the possibility of successful immune tolerance induction [147, 218]. Similarly, since multiple cytokines are coinstantaneously involved in the development or progression of autoimmunity, treatment with a complex of peptides from two or more different cytokines may have the potential to provide a more profound effect by concomitantly inhibiting those cytokines, which need to be evaluated in further studies.

Peptide-based vaccinations in rheumatic diseases are still at an early stage, and both the efficacy and safety of peptide-based vaccinations in patients with rheumatic diseases need to be validated in clinical trials. Besides, the optimal timing, dosing, and route of vaccinations also need be addressed before the initiation of its clinical use. Moreover, the role of prophylactic peptide-based vaccination in preventing or delaying the onset of rheumatic diseases among high-risk individuals is also of great interest and needs to be elucidated in future studies.

## Figures and Tables

**Table 1:** Comparison of those two peptide-based therapeutic vaccination strategies for rheumatic diseases.

Comparison items	Peptide-based active vaccination against pathogenic cytokines	Peptide-based tolerogenic vaccination
Sources of peptides	Pathogenic cytokines	Self-antigens, TCR repertoire
Therapeutic targets	Pathogenic cytokines	Autoimmune attacks against host cells or tissues caused by autoreactive lymphocytes
Main effects	Induce the production of neutralizing antibodies against pathogenic molecules	Induce immune tolerance to self-antigens by inhibiting autoreactive lymphocytes while promoting Tregs
Adjuvant	Need adjuvant	Not necessary
Relevant immune cells	Mainly B cells	Autoreactive T cells, Tregs, tolDCs, etc.
Evidence from clinical trials	Limited	Limited
Diseases	RA, SLE, and SjS	RA, SLE, and SjS

Tregs: regulatory T cells; tolDCs: tolerogenic dendritic cells; RA: rheumatoid arthritis; SLE: systemic lupus erythematosus; SjS: Sjögren's syndrome; VLP: virus-like particle; TCR: T cell receptor.
